# Assembly of gold nanoparticles into aluminum nanobowl array

**DOI:** 10.1038/s41598-017-02552-z

**Published:** 2017-05-24

**Authors:** Xingce Fan, Qi Hao, Renchao Jin, Hao Huang, Zhengwei Luo, Xiaozhi Yang, Yile Chen, Xingzhi Han, Meng Sun, Qihua Jing, Zhenggao Dong, Teng Qiu

**Affiliations:** 1 School of Physics, Southeast University, Nanjing, 211189 P.R. China; 2 School of Chemistry and Chemical Engineering, Southeast University, Nanjing, 211189 P.R. China

## Abstract

We mimic unique honeycomb structure as well as its functions of storing honey and pollen to assemble Au nanoparticle pattern on honeycomb-like Al nanobowl array by utilizing solid state dewetting process. Patterned Au nanoarrays of ‘one particle per bowl’ with tunable plasmonic bands ranging from the visible to the near-infrared region are fabricated by finely selecting the initial thickness of Au film, the geometry of Al nanobowl array and the thermal treatment parameters. This work presents a powerful approach to assemble Au nanoparticles into high density nanoarrays with superior spatial resolution, offering highly concentrated electromagnetic fields for plasmonic sensor applications.

## Introduction

In nature, honeycomb typically shows a closely packed hexagonal wax cells where honey and pollen are stored. This most efficient, least wasteful and strongest building system continues inspiring human to engineer honeycomb-like structures^1^. In nanometer world, we mimic honeycomb structures and its versatile functions to precisely assemble patterned Au nanoarrays on honeycomb-like substrates by means of a facile solid state dewetting (SSD) process^2–6^. SSD describes the formation of island particles when solid state matters (e.g. metals) are heated to sufficiently high temperatures (below melting temperature). This process can be used to manipulate metallic nanostructures with preset parameters by utilizing nanotemplates and optimizing the total surface energy of metallic thin film, substrate and their interfaces^2, 6–15^.

When a metallic thin film is dewetted on a nanotemplate, the formation of nanoisland array depends on the template structures. Hence, the pattern of template determines the spatial resolutions of dewetted metallic nanoarray. The spatial resolution controls the plasmonic characteristics of the metallic nanoarrays, which are critical for plasmonic sensor applications, such as surface-enhanced Raman scattering and surface-enhanced fluorescence^16–20^. Therefore, it is vital to seek for suitable nanotemplates with high spatial resolution, uniform unit size as well as tunable surface morphology to modulate the SSD process and subsequent nanopatterning.

Currently, common methods for nanotemplate fabrication, such as electron beam lithography^21, 22^, focused ion beam lithography^23, 24^, optical interference lithography^6, 9^ and colloidal lithography^11, 12^, can allow one to design the template morphologies. However, electron beam lithography and focused ion beam lithography are too expensive in practical applications, and optical interference and colloidal lithography are typically limited by the low spatial resolutions owing to the wavelength of optical sources and the size of colloidal spheres, respectively. Alternatively, titania nanotube^25–27^ and anodic aluminum oxide (AAO)^20, 28–30^ produced by electrochemical self-assembly approaches were typically employed as templates to rationally engineer metallic nanostructures with high spatial resolutions and density. However, the dewetted metallic nanostructures on titania nanotube or AAO may probably generate core-satellite nanostructures^20, 26, 28^ leading to exhibiting additional hybrid plasmonic modes among the visible to the near-infrared (NIR) region, which are not satisfying in high sensitive sensor applications^31^.

In this work, we induce initial instabilities in as-deposited Au film by using Al nanobowl array template to assemble patterned Au nanoarrays by SSD process. Compared with other templates discussed above, the bowl structures of template offers a much higher spatial resolution for the dewetted metallic nanoarray with uniform unit size. Besides, the Al nanobowl array presents a high structural flexibility regarding achievable bowl size ranging from ~50 to ~400 nm^32^. Herein, a series of Au nanoparticles can be well assembled into nanopatterns on ordered Al nanobowl array with ‘one particle per bowl’. The patterned Au nanoarrays exhibit high spatial resolutions and tunable plasmonic bands over a broad range from the visible to the NIR region.

## Results and Discussion

**Figure 1:**
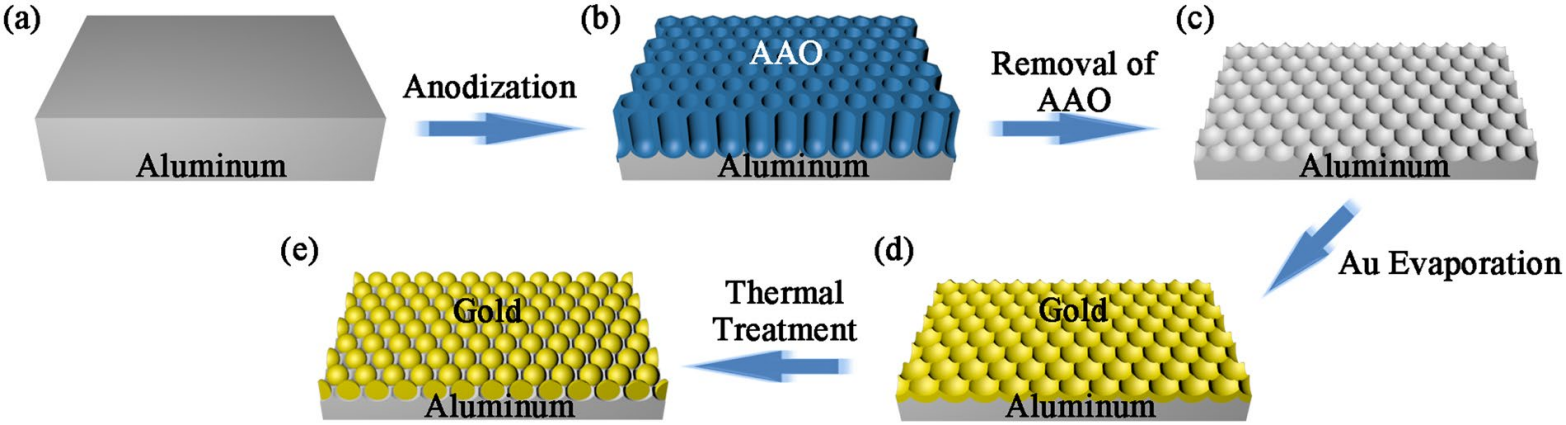
Schematic illustrations of the preparation of Al nanobowl array template and subsequent SSD process for Au nanoparticles assembly: (**a**) pretreated Al foil, (**b**) AAO template after anodization, (**c**) Al nanobowl array template after removal of AAO, (**d**) the Au-coated Al nanobowl array after thermal deposition, (**e**) Au nanoparticle array assembled by SSD process.

The assembly of Au nanoparticles into honeycomb-like Al nanobowl array by SSD process is schematically shown in Fig. 1, which starts with the fabrication of AAO template. After removal of AAO layer, the underlying honeycomb-like Al nanobowl array was employed as topographic template in this work.

**Figure 2:**
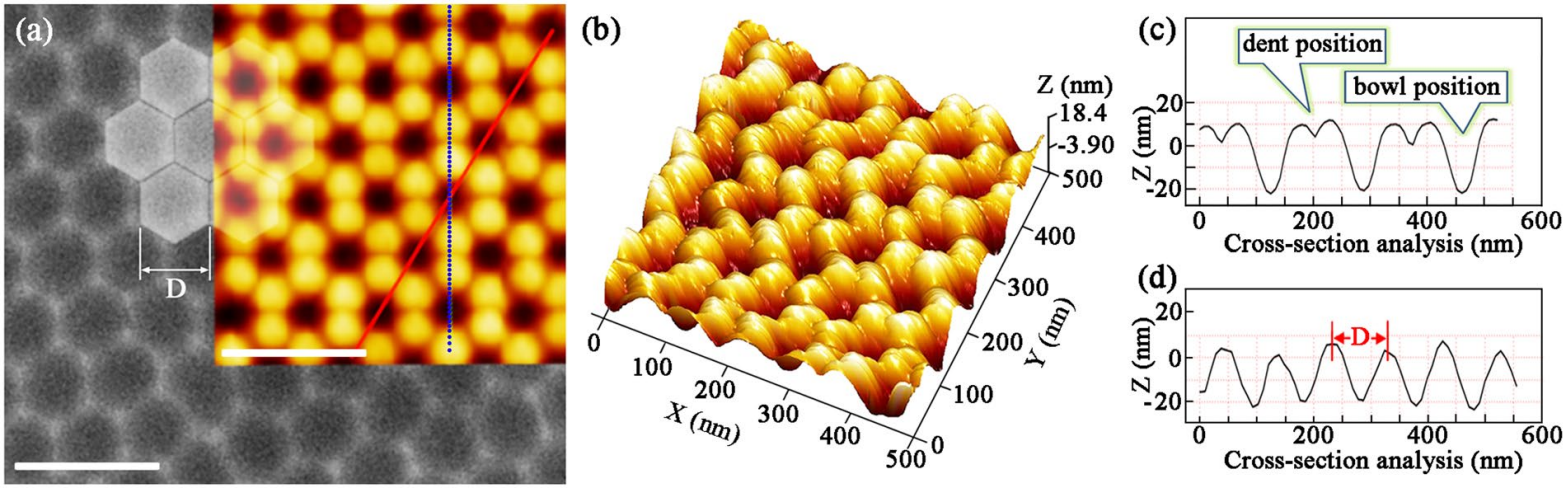
(**a**) A typical SEM image of Al nanobowl array (*V*_AAO_ = 40 V) and the inset is a 2D AFM image. The scale bars are both 200 nm. (**b**) 3D AFM image of the Al nanobowl array (*V*_AAO_ = 40 V). (**c,d**) Cross-section analysis along the dash and solid lines in the inset of (**a**), respectively.

The SEM image and the high-resolution 2D AFM image of Al nanobowl array (*V*_AAO_ = 40 V) are illustrated in Fig. 2a and its inset respectively, where the hexagonal units are clearly marked. Each Al nanobowl is surrounded by six equivalent adjacent ones and can easily scale up to contiguous area of centimeter dimension. The high resolution 3D AFM image of the same Al nanobowl array in Fig. 2b depicts that there are six small protrusions along each nanobowl (named as bowl position), as well as a dent exists between every two adjacent protrusions which all exhibit concave shape (named as dent position). To describe this surface morphology more clearly, cross-section analysis along the dash and solid lines in the inset of Fig. 2a are conducted. The corresponding results are present in Fig. 2(c,d), in which top-to-bottom fluctuations along the unit boundary between adjacent two protrusions and the diagonal between dents are obvious. The *D* value of each nanobowl is ~100 nm. Moreover, the average D value can be tailored from 60 nm to 150 nm by adjusting the *V*_AAO_ value from 20 V to 60 V, and the functional relationship between *D* and *V*_AAO_ is approximately linear^33–35^.

**Figure 3:**
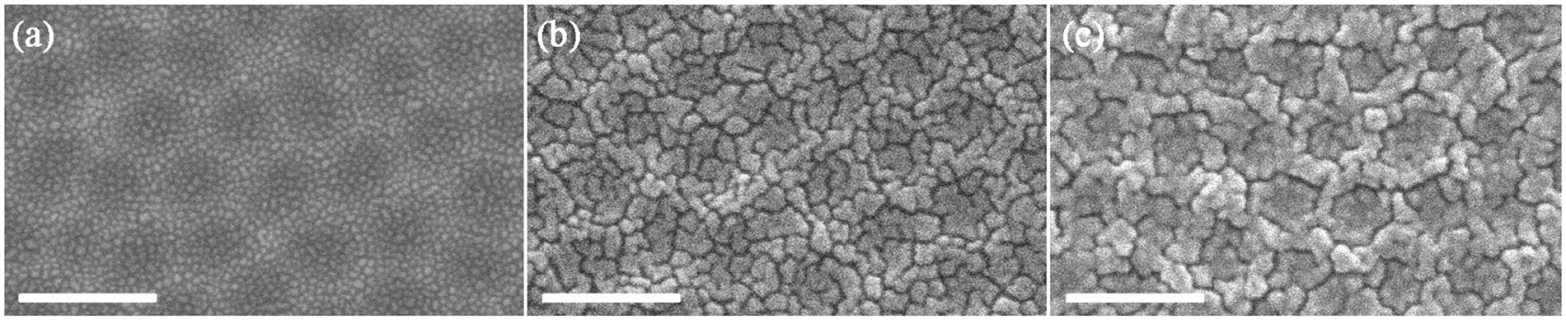
SEM images of Au-coated Al nanobowl arrays (*V*_AAO_ = 40 V) with *t*_Au_ value of (**a**) 2.5 nm, (**b**) 7.5 nm and (**c**) 12.5 nm, respectively. The scale bars in SEM images are 200 nm.

Then, thin layers of Au are thermally evaporated onto the Al nanobowl arrays (*V*_AAO_ = 40 V) with different thicknesses as shown in Fig. 3. Although the thickness of Au film increases, the honeycomb-like units are still visible. The as-deposited Au films all exhibit cracking surface morphologies due to the local stress induced by Al nanobowl array^36, 37^ and the character of thermal evaporation method, which both facilitate the subsequent SSD process dramatically. However, it is worth to note that the Au film (*t*_Au_ = 2.5 nm) is quite different from the other two ones, exhibiting discontinuous cracking morphology. According to previous study^6^, SSD process is typically initiated at the edge of thin film or defect sites. Hence, the discontinuous and continuous Au film are supposed to display different results after SSD process^12^.

**Figure 4:**
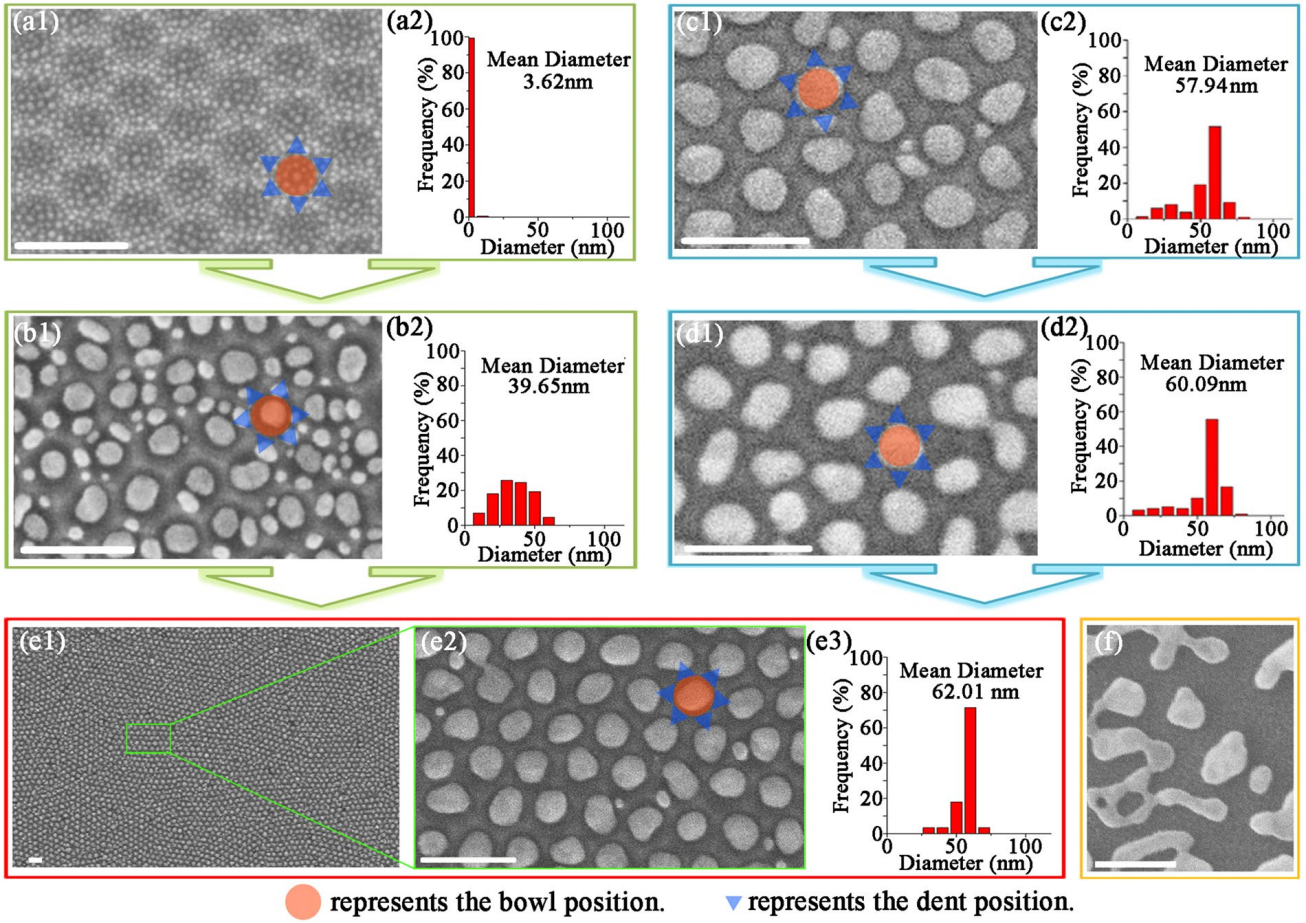
Structural evolutions after SSD process (400 °C for 2 h) for Au-coated Al nanobowl array (*V*_AAO_ = 40 V) with different Au film thickness: (**a1**) 2.5 nm, (**b1**) 7.5 nm and (**e2**) 12.5 nm. Structural evolutions for Au-coated Al nanobowl array (*t*_Au_ = 12.5 nm, *V*_AAO_ = 40 V) with different thermal treatment temperatures during SSD process: (**c1**) 300 °C for 2 h and (**d1**) 350 °C for 2 h. (**e1**) Large-scale SEM image corresponding to (**e2**). (**a2–d2**) and (**e3**) are corresponding calculated diameter distributions in (**a1–d1**) and (**e2**), respectively. (**f**) Au nanoparticles acquired under the same conditions as in (**e1**) without using Al nanobowl array template. The scale bars in SEM images are all 200 nm.

When the Au-coated Al nanobowl arrays undergo thermal treatment, the Au films start to interact with the topography of underlying Al nanobowl arrays as shown in Fig. 4. The dewetting result shown in Fig. 4(a1) proves that the numerous pre-existed cracks in discontinuous Au film will behave as nuclei during the thermal treatment^37, 38^. As the SSD proceeds, they grows larger and leads to uniformly located Au nanoparticles on the inner surface of the Al nanobowl with narrow diameter distribution and small interpaticle distance of about 5 nm. These interstitial sites are believed to have highly concentrated electromagnetic fields associated with strong localized surface plasmon resonance (LSPR)^39^.

Different from dewetted discontinuous Au film, SSD process of thicker Au film (Fig. 4(b1) and (e1)) begins with grooving of the Au film at the edge of nanobowls^3^ and cracks in the Au film, resulting in subsequent SSD process mainly takes place at each bowl position independently. It should be noted that the dent position between adjacent protrusions also hold small amount of Au, which also endures a SSD process during the thermal treatment, leading to the formation of the small patches surrounding the nanobowl (Fig. 4(b1)). However, when the thickness of Au film increases to 12.5 nm, the dewetted Au nanoparticles at dent position nearly disappear, and an array of ‘one particle per bowl’ was acquired, which results from the direct contact of Au nanoparticles at dent position and bowl position. In general, atomic diffusion progresses from smaller particle to the largest, thus causing the Au nanoparticles at the dent position to diffuse to the bowl position^11^. The corresponding narrow size distribution of dewetted Au nanoparticles shown in Fig. 4(e3) also verifies that patterned Au nanoparticles can be assembled on highly ordered Al nanobowl array template. The corresponding normalized extinction spectra of dewetted Au nanoparticle arrays with different *t*_Au_ values are shown in Figure S1(a), which clearly reveal that the red-shift of plasmonic bands are in agreement with the statistical size distributions. Besides, we find that the Au nanoparticles at the bowl position nearly reach the size limit (the confinement of Al nanobowl) at this case, and much thicker Au film is excessed relative to the Al nanobowl depth, resulting in the dewetted Au nanoparticles at bowl positions contact each other through dent position (as shown in Figure S2). This phenomenon indicates there is a suitable Au film thickness for a certain Al nanobowl array template for realizing ‘one particle per bowl’ array.

The driving force behind the formation of ‘one particle per bowl’ array can be explained by the theory of surface energy minimization^36, 40, 41^. The Gibbs-Thomson relation^6^ $\Delta \mu =\kappa \gamma \Omega$, where *Δμ* is the local excess chemical potential, $\kappa$ is the local curvature, $\gamma$ is the surface energy and $\Omega$ is the atomic volume, allows the Au atoms diffusing away from the protrusion to the bowl and dent position to reduce surface energy and local curvature, leading to the decreasing of the local excess chemical potential. Reference sample was thermally treated in the same way but using flat Al template, only exhibiting disordered nanoparticles (Fig. 4f), which further underlines the local curvature-related surface morphologies of template playing a significant role in the formation of well-controlled Au nanoparticle array. By comparison, SSD on Al nanobowl array template causes a significant reduction in Au nanoparticle size compared to on flat one, which is of great significance in realizing high spatial resolutions in this work.

The rate of SSD is strongly temperature dependent, because dewetting behavior requires atomic transport^2^. Previous study has demonstrated that the different thermal treatment temperatures may result in totally different surface morphologies, and the available thermal treatment temperature for thin Au film was estimated to approximately 128 °C to 496 °C by using Tammann and Huttig temperatures^11^. Figure 4(c1), (d1) and (e2) illustrate typical dewetting results of Au-coated Al nanobowl array (*t*_Au_ = 12.5 nm, *V*_AAO__=_ 40 V) with different thermal treatment temperatures: 300 °C, 350 °C and 400 °C, respectively. At lower thermal treatment temperature (300 °C and 350 °C), there are relatively more small Au nanoparticles existing at the dent position compared with 400 °C. The average diameter of Au nanoparticles also rises with increasing of thermal treatment temperature, indicating the small Au nanoparticles at dent position diffuse to the bowl position (Fig. 4(c2),(d2)). The corresponding normalized extinction spectra of Au nanoparticle arrays with different thermal treatment temperatures are shown in Figure S1(b), which are in agreement with the statistical results. Our experiment results indicate that relatively high temperature is desirable for realizing the array of ‘one particle per bowl’.

As aforementioned, we can acquire Al nanobowl array with tunable unit size *D* from 60 nm to 150 nm by increasing the *V*_AAO_ value from 20 V to 60 V. The functional relationship between *V*_AAO_ and *D* ^33^ is given by:

1 $$D=2.5\,{V}_{AAO}$$

**Figure 5:**
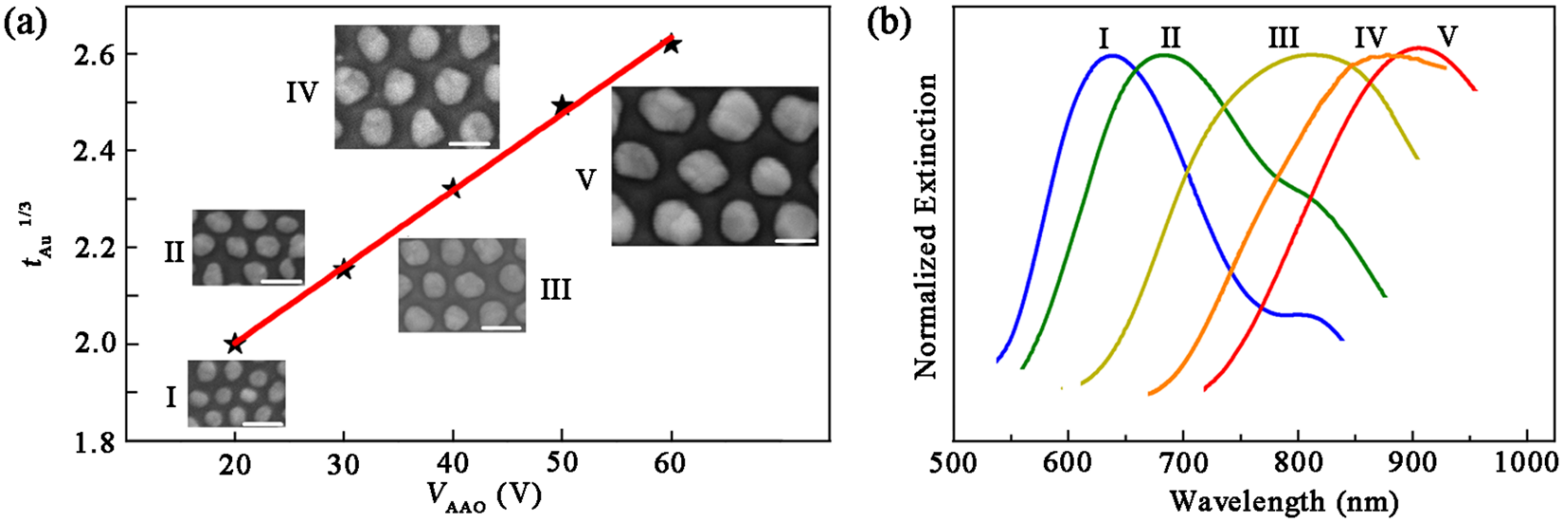
(**a**) The empirical relationship between *V*_AAO_ and *t*_Au_ based on experiment results. The insets in (**a**) give the corresponding SEM images of ‘one particle per bowl’ array with different *V*_AAO_ values. (**b**) Normalized extinction spectra corresponding to the inset images in (**a**). I: *V*_AAO_ = 20 V, *t*_Au_ = 8.0 nm, thermal treatment for 1 h; II: *V*_AAO_ = 30 V, *t*_Au_ = 10.0 nm, thermal treatment for 2 h; III: *V*_AAO_ = 40 V, *t*_Au_ = 12.5 nm, thermal treatment for 2 h; IV: *V*_AAO_ = 50 V, *t*_Au_ = 15.0 nm, thermal treatment for 3 h; V: *V*_AAO_ = 60 V, *t*_Au_ = 18.0 nm, thermal treatment for 4 h. The scale bars in the inset of (**a**) are 100 nm.

In this work, we realized the assembly of Au nanoparticles into different Al nanobowl array with ‘one particle per bowl’ as shown in the insets of Fig. 5a. The suitable thicknesses of as-deposited Au films are summarized in Table 1. We find that the empirical relationship between *V*_AAO_ and *t*_Au_^1/3^ is approximately linear as shown in Fig. 5a. It is reasonable to assume that the dewetted Au nanoparticles are all spheres with the diameter value of *d*, which can be calculated by function:

2 $$\frac{\sqrt{3}{D}^{2}{t}_{{Au}}}{2}=\frac{4}{3}\pi {(\frac{d}{2})}^{3}$$

**Table 1:** Experimental and calculated results for realizing ‘one particle per bowl’ array.

*V*_AAO_ (V)	20	30	40	50	60
*D* (nm)	60	75	100	125	150
*t*_Au_ (nm)	8.0	10.0	12.5	15.0	18.0
Mean Diameter of Au Nanoparticles (nm)	44.27 ± 6.86	54.37 ± 9.64	62.07 ± 7.12	81.40 ± 13.26	107.95 ± 21.01
Calculated Au Nanoparticle Diameter (nm)	36.25	45.31	59.13	72.91	87.50
Densities of Au nanoparticles (10^10^ cm^−2^)	3.21	2.05	1.15	0.739	0.513

The calculated results are also summarized in Table 1. Compared with calculated results, we find that the experimental results are slightly larger, which may results from the fact that Au nanoparticles are not perfect spheres and there is a large interface between the particles and underlying Al nanobowl^11^.

The plasmonic properties of patterned Au nanoarrays were also experimentally investigated. The extinction spectra of Au nanoarrays shown in Fig. 5b are generated from the LSPR of Au nanoparticles, which is a phenomenon that the incident light directly couples to resonant free electron in Au nanoparticles, only occurring at a particular frequency of the incident light^18, 39, 42^. It is known that the plasmonic bands of nanoparticles can be tuned by changing the particle diameters. In this work, the Au nanoparticle diameters can be designed by finely selecting Al nanobowl array templates and suitable Au film thickness. The variations in diameter result in shifts of plasmonic bands (Fig. 5b). The redshifts originate from the phase delay across the nanoparticles^43^. The extinction peak at ~810 nm for curve I and II can be ascribed to interband transition of underlying Al nanobowl array^44^. Our results indicate that SSD process on Al nanobowl array template presents distinct capability in tuning plasmonic bands over a broad range from the visible to the NIR region, which allows one tuning plasmonic bands depending on the availability of a suitable laser or to resonantly enhance an optical process, such as surface-enhanced Raman scattering and surface-enhanced fluorescence.

## Conclusion

In summary, this work presents a powerful approach to assemble Au nanoparticles into high density nanoarrays with superior spatial resolution by employing electrochemically fabricated Al nanobowl array templates and facile SSD process. By well controlling the initial Au film thickness, the geometry of Al nanobowl array template and the thermal treatment parameters, a series of ordered Au nanoarrays of ‘one particle per bowl’ is successfully fabricated. Their plasmonic properties are experimentally investigated and plasmonic bands can be tuned over a broad range from the visible to the NIR region. The patterned Au nanoarrays fabricated by this technique has potential applications in plasmonic sensor applications.

## Methods

### Synthesis of Al nanobowl array templates

High purity Al foils (99.999%, 30 mm × 30 mm × 1 mm) were firstly degreased by acetone and deionized water, and then electro-polished by using a mixture of ethanol and perchloric acid with volume ratio of 4:1 under a constant direct-current voltage of 15 V for 3 min to further remove the surface impurities. After rinsing in deionized water and drying, the Al foils were anodized separately in a 0.3 M oxalic acid solution at 4 °C at a constant direct-current voltage (denoted as *V*_AAO_) of 20 V (30 V, 40 V, 50 V and 60 V). In order to obtain highly ordered Al nanobowl arrays, a two-step electrochemical anodizing process was adopted^30, 45^. The Al foils were first anodized for 2 h followed by immersion into a mixture of chromic acid (1.8 wt%) and phosphoric acid (6 wt%) at 75 °C (1:1 in volume) for 2 h. The AAO layer which grew during the first step was removed and the surface of the Al foil became bright again. The second anodizing step and AAO removal process were carried out the same as the first step. Finally, Al nanobowl array templates with different unit sizes (denoted as *D*) can be obtained.

### Solid state dewetting process of Au films

The Au film evaporation procedure was conducted in a Thermal Evaporation System (ZHD-400), and the film thickness (denoted as *t*_Au_) was monitored by Film Thickness Monitor (Taiyao FTM-V). Au films with different *t*_Au_ values were thermally evaporated onto the Al nanobowl array templates and the evaporation rates were all maintained at ~0.01 nm s^−1^ with initial chamber pressure below 2 × 10^−4^ Pa. Next, thermal treatments were conducted at 400 °C (300 °C or 350 °C) for sufficient times under a pressure of ~0.1 Pa in a CVD furnace, and followed by rapid cooling in air.

### Instrumentation and data acquisition

Atomic Force Microscopy (AFM) (BRUKER Dimension Icon) was used to investigate the surface morphology of Al nanobowl array template. Field Emission Scanning Electron Microscopy (FE-SEM) (FEI Inspect F50) was employed to observe the morphological evolutions of the surface nanostructures under different synthesis parameters. The UV-Vis spectra were acquired from a SHIMADZU UV-2600 Spectrophotometer. Image-Pro Plus Version 6.0 was used to calculate the Au nanoparticle diameter distributions.

## Electronic supplementary material

Supplementary Info
