# Supplementary material for: Assembly of gold nanoparticles into aluminum nanobowl array
Source: Sci Rep. 2017 May 24;7:2322. doi: 10.1038/s41598-017-02552-z (PMC5443813; doi:10.1038/s41598-017-02552-z)
Supplement: Supplementary file 1 — Supplementary Info [file 41598_2017_2552_MOESM1_ESM.pdf]

Supporting Information

Assembly of gold nanoparticles into aluminum nanobowl array

Xingce Fan[1], Qi Hao[1], Renchao Jin[1], Hao Huang[1], Zhengwei Luo[1], Xiaozhi Yang[1], Yile Chen[2], Xingzhi Han[1], Meng Sun[1], Qihua Jing[1], Zhenggao Dong[1] and Teng Qiu[1,*]

[1] School of Physics, Southeast University, Nanjing 211189, P. R. China.

[2] School of Chemistry and Chemical Engineering, Southeast University, Nanjing 211189, P. R. China.

Email: tqiu@seu.edu.cn (T. Qiu)

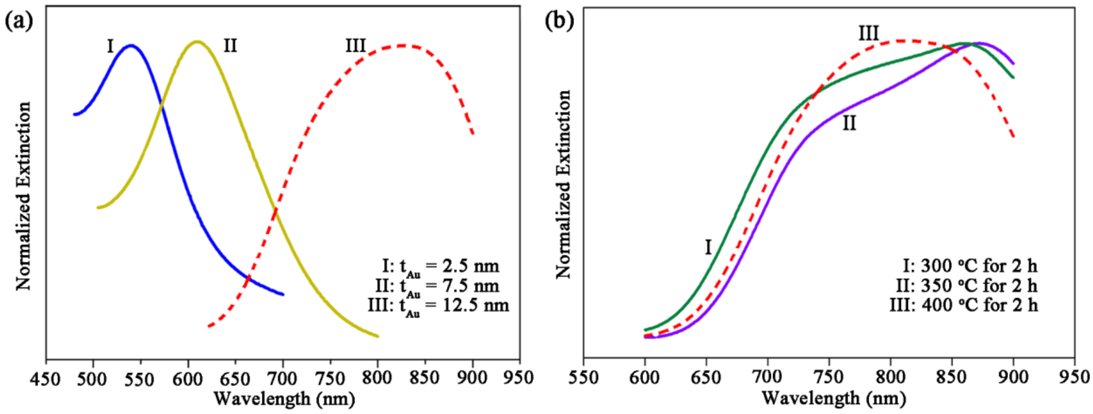

Figure S1. (a) Normalized extinction spectra of Au-coated Al nanobowl array (V_{AAO} = 40 V) after SSD process (400 ºC for 2 h) with different t_{Au} values, curve I: 2.5 nm and curve II: 7.5 nm. (b) Normalized extinction spectra of Au-coated Al nanobowl array (t_{Au} = 12.5 nm, V_{AAO} = 40 V) after SSD process with different thermal treatment temperatures, curve I: 300 ºC for 2 h and curve II: 350 ºC for 2 h. The dash curve shown in (a) and (b) are the normalized extinction spectra from the sample shown in Figure 4(e1).

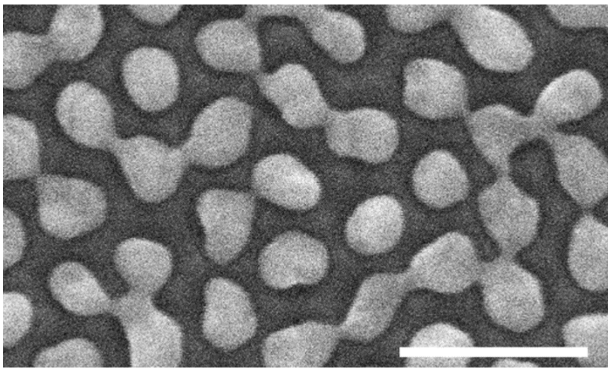

Figure S2. SEM image of Al nanobowl array (V_{AAO} = 40 V) with excessive amount of Au (t_{Au} = 15.0 nm) after SSD process (400 ºC for 3 h). The scale bar is 200 nm.
